# 

**Published:** 1892-09

**Authors:** 


					﻿Vol. XII.
SEPTEMBER, 1892.
NO. 9.
THE
JIOMtEOPATHIC pHY^lClAJfl,
A Monthly Journal of Homoeopathic Materia Medica
and Clinical Medicine.
"He is afreeman whom truth makes free, and all are slaves beside."
"Seek the Truth: come whence it may, cost what it will."
EDITED AND PUBLISHED BY
WALTER XI. JAMES, XT. D.
PHILADELPHIA :
No. 1125 SPRUCE STK1CICT.
LONDON:
ALFRED HEATH & CO’., 8oi.b Agemts fob Great Britain,
114 Ebury Street, Eaton Square.
93~Tearly Subscription, $2.50. invariably in, advance. Single numbers, 25 els.
Entered at the Philadelphia Post-Office aa Second Class Mall Matter.
				

